# Evaluation of the Effects of Switching COPD Patients From LAMA/LABA Therapy to ICS/LAMA/LABA Therapy Using the Impulse Oscillation System (IOS) Capable of Separating Inspiratory and Expiratory Measurements

**DOI:** 10.1111/crj.70105

**Published:** 2025-07-15

**Authors:** Yosuke Tanaka, Ken Okamura, Shota Kaburaki, Toru Tanaka, Akiko Yoshikawa, Ayumi Shimizu, Akihiko Miyanaga, Namiko Taniuchi, Koichiro Kamio, Kazuo Kasahara, Masahiro Seike, Mitsunori Hino

**Affiliations:** ^1^ Department of Pulmonary Medicine and Oncology, Graduate School of Medicine Nippon Medical School Tokyo Japan

**Keywords:** airway resistance, COPD, impulse oscillation system (IOS), inhaled corticosteroid (ICS), MostGraph

## Abstract

**Introduction:**

Noninvasive evaluation of airway conditions is considered useful in the management of COPD, although assessing airway remodeling remains difficult in routine clinical practice. The impulse oscillometry system used in this study allows separate analysis of inspiratory and expiratory phases, offering detailed insights into airway function. This study examined the effects of inhaled corticosteroids (ICSs) on airway remodeling and assessed the utility of this system in COPD management.

**Methods:**

Stable COPD patients on LAMA/LABA for over a year were assessed by spirometry and impulse oscillometry at baseline and after 48 weeks of ICS/LAMA/LABA therapy. Symptoms, imaging, and blood tests were also evaluated.

**Results:**

Among 52 patients (mean baseline %FEV1/predicted: 56.9% ± 22.1%), all had one to two moderate exacerbations in the past year despite LAMA/LABA therapy. Significant correlations were observed between spirometry and MostGraph (e.g., baseline FEV1 vs. R5: *r* = −0.54). Although spirometry showed no significant changes, Fres improved significantly (−2.11 ± 0.35, *p* < 0.0001), with reductions in both expiratory and inspiratory phases.

**Conclusions:**

Fres measured by MostGraph significantly improved after ICS addition, whereas no significant changes were observed in spirometry or resistance parameters. Fres also showed significant correlations with FEV1, suggesting that it may capture airway changes not detected by spirometry. These findings support further investigation into its role as a noninvasive marker in COPD.

**Trial Registration:**

UMIN‐CTR Clinical Trial: UMIN000040764 (https://upload.umin.ac.jp/cgi‐open‐bin/ctr_e/ctr_view.cgi?recptno=R000042394)

## Graphical abstract

This study assessed the effect of switching from dual bronchodilator therapy to triple therapy in COPD. While FEV1 showed no significant change, resonant frequency improved, suggesting reduced airway inflammation. Impulse oscillometry may help detect treatment effects not captured by spirometry.

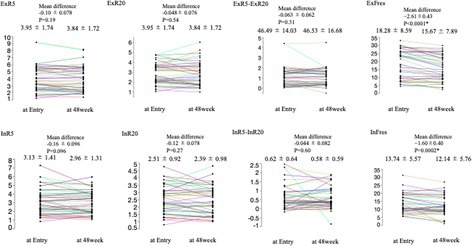

## 1 Introduction

Chronic obstructive pulmonary disease (COPD), asthma, and asthma–COPD overlap (ACO) are chronic respiratory diseases characterized by shared symptoms and pathophysiological features in the bronchi [1]. These diseases exhibit both “complexity,” defined by the coexistence of diverse and interrelated clinical features, and “heterogeneity,” which refers to the distinct clinical presentations unique to individual patients. This highlights the need for personalized assessment and management based on differences in treatment responses [2, 3, 4]. Traditionally, the evaluation and management of COPD have relied on the severity of airflow limitation. However, there is a growing recognition of the importance of addressing the disease's complexity and heterogeneity more comprehensively [2]. Recent guidelines have proposed an integrated approach that incorporates multiple variables, emphasizing the concept of “treatable traits” to identify patient‐specific characteristics and phenotypes [2, 3, 4]. Treatable traits identified at the molecular level hold significant promise for advancing personalized medicine, though further research is needed. Applying this concept to clinical practice also requires identifying treatable traits in COPD and other chronic airway diseases, along with achieving an international consensus on treatment strategies [5]. Previous studies have shown mixed results regarding the effectiveness of inhaled corticosteroids (ICSs) in COPD. Some reports indicate beneficial effects of ICS, such as reduced exacerbations and improved symptoms or lung function [6, 7, 8, 9, 10, 11], whereas others highlight limited efficacy or potential risks [12, 13]. Additionally, recent large‐scale randomized controlled trials, such as the IMPACT and ETHOS studies, have demonstrated that triple therapy (ICS/LABA/LAMA) more effectively reduces exacerbation frequency and improves lung function than dual therapies (ICS/LABA or LAMA/LABA), particularly in patients with frequent exacerbations or eosinophilic inflammation [14, 15]. Although these findings support the use of ICS in selected COPD phenotypes, it is important to note that our study specifically excluded patients with high blood eosinophil counts or clinical features suggestive of asthma. By applying strict entry criteria, we aimed to evaluate the therapeutic effects of ICS‐based triple therapy in patients with airway lesions primarily attributable to COPD rather than overlapping inflammatory conditions.

The introduction of the ACO concept has further complicated the assessment of ICS efficacy, as determining which patients benefit from ICS, the appropriate timing for initiation, and when to discontinue treatment remain major challenges. In clinical practice, particularly in elderly patients, a less invasive method for assessing airway inflammation and structural changes is urgently needed. Although pulmonary function tests are commonly used to evaluate airway obstruction, their reliance on adequate inspiratory and expiratory maneuvers often makes them challenging for elderly patients, hindering accurate assessments of advanced airway remodeling. Impulse oscillometry system (IOS) parameters, including the resonance frequency (Fres), have been proposed as noninvasive indicators of airway mechanics in COPD. Fres, which reflects the balance between elastic and inertial resistance, may offer potential as a surrogate marker for airway remodeling. However, this remains a hypothesis requiring further validation through imaging or histological evaluation [16].

Although ICS lacks direct bronchodilatory effects, previous studies have demonstrated modest improvements in FEV1, particularly in patients with eosinophilic inflammation or asthma–COPD overlap, potentially due to anti‐inflammatory and vasoconstrictive effects. However, in this study, we aimed to minimize the influence of such asthma‐related traits by excluding patients with evident asthmatic features based on predefined entry criteria, thereby focusing the evaluation on airway responses in patients with predominantly COPD pathology.

The FLAME trial demonstrated that LAMA/LABA therapy reduced exacerbation rates more effectively than ICS/LABA therapy [12]. The WISDOM trial reported no significant difference in the time to the first exacerbation after ICS withdrawal during triple therapy, although lung function declined more rapidly following ICS withdrawal [13]. Despite limited direct effects on airway obstruction, these findings suggest that ICS might exert suppressive effects on airway remodeling in certain patients.

Evaluating airway remodeling using only conventional pulmonary function tests requires frequent, detailed assessments over short intervals to monitor the progression of airway obstruction. However, this approach only confirms airway remodeling after significant obstruction has already occurred, making it challenging to capture subtle pathological changes in remodeling.

If a reliable marker were available to assess the effects of ICS on airway remodeling independent of improvements in airway obstruction, it could provide valuable clinical insights. Such a marker could help identify patients who are likely to benefit from ICS in targeting airway remodeling, determine whether a given patient has remodeling‐related lesions that are amenable to ICS therapy, and establish the optimal timing for initiating ICS. Furthermore, it could guide decisions on when to discontinue ICS after the therapeutic effects on remodeling have been achieved or to restart ICS if remodeling progression resumes following its withdrawal.

Having a practical and reliable tool to evaluate airway remodeling would greatly aid in tailoring ICS therapy to individual patients, optimizing treatment timing, and improving overall clinical management of airway remodeling.

In Japan, ICSs are commonly prescribed for COPD patients with persistent symptoms despite bronchodilator therapy, provided there are no contraindications. Managing airway lesions, including remodeling, requires a thorough understanding of the disease's pathophysiology as well as the appropriate timing for initiating and discontinuing ICS therapy.

To address these challenges, we hypothesized that the MostGraph could serve as a noninvasive tool for assessing ICS‐induced changes in airway remodeling. Building on our previous findings [14], we examined the reproducibility of the association between MostGraph parameters and airway obstruction. Specifically, we investigated the correlation between conventional pulmonary function tests and MostGraph‐derived indices, such as total airway resistance (R5), central airway resistance (R20), and peripheral airway resistance (R5–R20). ICS, which lacks a bronchodilatory effect, produced minimal changes in these resistance parameters (R5, R20, and R5–R20), but significant changes were observed in the resonance frequency (Fres), an index reflecting the balance between elastic and inertial resistance, which showed significant improvement following ICS treatment. These findings suggest that although ICS does not directly affect airway resistance, it may lead to changes in airway mechanics suggestive of remodeling, although further validation is required.

This study aimed to investigate changes in airway functional markers, including oscillometric and spirometric indices, in patients with COPD following the initiation of ICS as part of triple therapy. In particular, we examined the potential responsiveness of Fres and its correlation with conventional lung function parameters.

## 2 Participants

Study Period: April 15, 2021–July 31, 2027

Enrollment Period: April 15, 2021–July 31, 2026

We enrolled stable patients aged ≥ 40 years who had been diagnosed with COPD and received LAMA/LABA therapy for over 1 year. Patients with features suggestive of asthma (e.g., history of asthma, bronchodilator reversibility, and eosinophilia), other pulmonary diseases, or prior ICS/LABA/LAMA use were excluded.

These criteria were established based on the 2nd edition of the Asthma and COPD Overlap (ACO) guidelines by the Japanese Respiratory Society to ensure the evaluation focused on COPD pathology. Full inclusion/exclusion criteria are provided in the Supporting Information (Entry Criteria).

## 3 Study Treatment

Patients with COPD who have been receiving LAMA/LABA therapy for > 12 months will be switched to ICS/LAMA/LABA therapy for 12 months (Supporting Information [Treatment Methods]).

## 4 Ethics

The study was approved by the Medical Ethics Committee of Nippon Medical School. All the participants provided written informed consent.

## 5 Target Sample Size: 50 Cases

The target sample size of 50 cases was determined based on the interim analysis of our previous study [16], which examined changes in airway resistance using MostGraph during the transition from LAMA/LABA to ICS/LAMA/LABA. Clinical trial registration was completed before the trial began. Prior to patient recruitment, potential candidates were identified from our facility's records to prepare for enrollment. The trial was designed as a prospective study to verify the reproducibility of the final results from a previous study, which served as the basis for interim analysis. The sample size was chosen because of the rarity of IOS‐based studies in COPD treatment, the applicability of the paired‐samples *t*‐test for sample sizes exceeding 30, and the crossover design of the trial. Before starting patient enrollment, we re‐evaluated the final results from the previous study, which showed an expected change in the Fres value of 2.0, with a standard deviation of 1.0, significance level (*α*) of 0.05, and power (1 − *β*) of 0.8. This confirmed that the sample size was adequate, ensuring appropriateness before the start of patient enrollment.

### 5.1 Assessments (Please Refer to the Supporting Information [“Assessment”])

Pulmonary function was assessed using both spirometry and impulse oscillometry (IOS) with MostGraph at baseline and after 48 weeks of ICS/LAMA/LABA therapy.

Clinical assessments included the COPD Assessment Test (CAT) and modified Medical Research Council (mMRC) dyspnea scale, both evaluated at the same time points.

Additional evaluations included blood tests, imaging (chest X‐ray and chest CT), and treatment history.

Adverse events (onset date, type, severity, outcome, and outcome date) were monitored throughout the study. Details on the management of adverse events are provided in the Supporting Information (“Assessment,” “Handling of Adverse Events,” and “Management of Adverse Events”).

### 5.2 Primary Endpoint

Longitudinal changes in MostGraph 48 weeks after switching to ICS/LAMA/LABA therapy.

### 5.3 Secondary Endpoints

Changes before and after each drug therapy using data, including cases discontinued during the trial with all indicators.

### 5.4 Safety Assessment

Blood tests as needed; treatment information: dates and doses of various medication changes, steroids, and other medication status; adverse events: onset date, type, severity, outcome, and outcome date; clinical events: onset date, type, severity, outcome, and outcome date; prognostic information: outcome, outcome date, and cause of death.

Please refer to the Supporting Information “Handling of Adverse Events” for details on the management of adverse events.

### 5.5 *MostGraph

Respiratory impedance was assessed using a commercially available oscillatory system (MostGraph‐22 [Rev.1.2], Chest M.I. Co. Ltd., Tokyo, Japan), which has fulfilled standard recommendations, as described by Shirai et al. [17, 18]. The cheeks of the participants were supported while sitting and wearing a nose clip. They were then instructed to breathe quietly at the functional residual capacity level (tidal breathing) for approximately 30 s. The measurements were repeated until five technically acceptable records were obtained. Respiratory impedance was automatically calculated via a fast Fourier transformation using a personal computer with airflow and pressure signals in the mouth of the participants. The respiratory system resistance values at 5 and 20 Hz (R5 and R20, respectively), difference between R5 and R20 (R5–R20), respiratory system reactance at 5 Hz (X5), Fres, and low‐frequency reactance area (ALX) were evaluated. Each oscillatory index was expressed as the mean value during one respiratory cycle (whole breath), inspiratory and expiratory phases, and the difference between inspiratory and expiratory phases.

Eligible patients who had been receiving LAMA/LABA therapy for more than 1 year were switched from LAMA/LABA to ICS/LAMA/LABA within the period from the day all relevant parameters were evaluated 2 weeks later.

The evaluation 48 weeks after switching to ICS/LAMA/LABA therapy was conducted within the period from 48 to 49 weeks after the switch. Pulmonary function tests and impulse oscillometry (IOS) using MostGraph were performed when the study drug concentration was assumed to be at its trough level. Because MostGraph is a test conducted under normal breathing conditions, to avoid potential influences on the results of IOS, the IOS test using the MostGraph device was performed on the same day before the pulmonary function test.

## 6 Statistical Analysis

Unless consent is withdrawn by the study subjects, all clinical information of the study subjects will be included in the analysis.

Both effectiveness and safety were analyzed across all observation and examination items. Safety will be assessed in terms of the time to occurrence from medication and severity.

Changes in indicator items and changes in each group will be compared using Spearman's correlation test, and changes in numerical values of evaluation items over 1 year will be analyzed using the paired‐samples *t*‐test.

Evaluation of adverse events will involve comparing occurrence rates using the *χ* ^2^ test or Fisher's exact test, assessing the time to onset using the log‐rank test, and examining nonparametric tests and proportional hazard models.

For pulmonary function tests, following the IMPACT trial [9], a clinically meaningful change in FEV1 will be applied, using a minimum important difference of 15% or more.

## 7 Results

### 7.1 Patients

**FIGURE 1:**
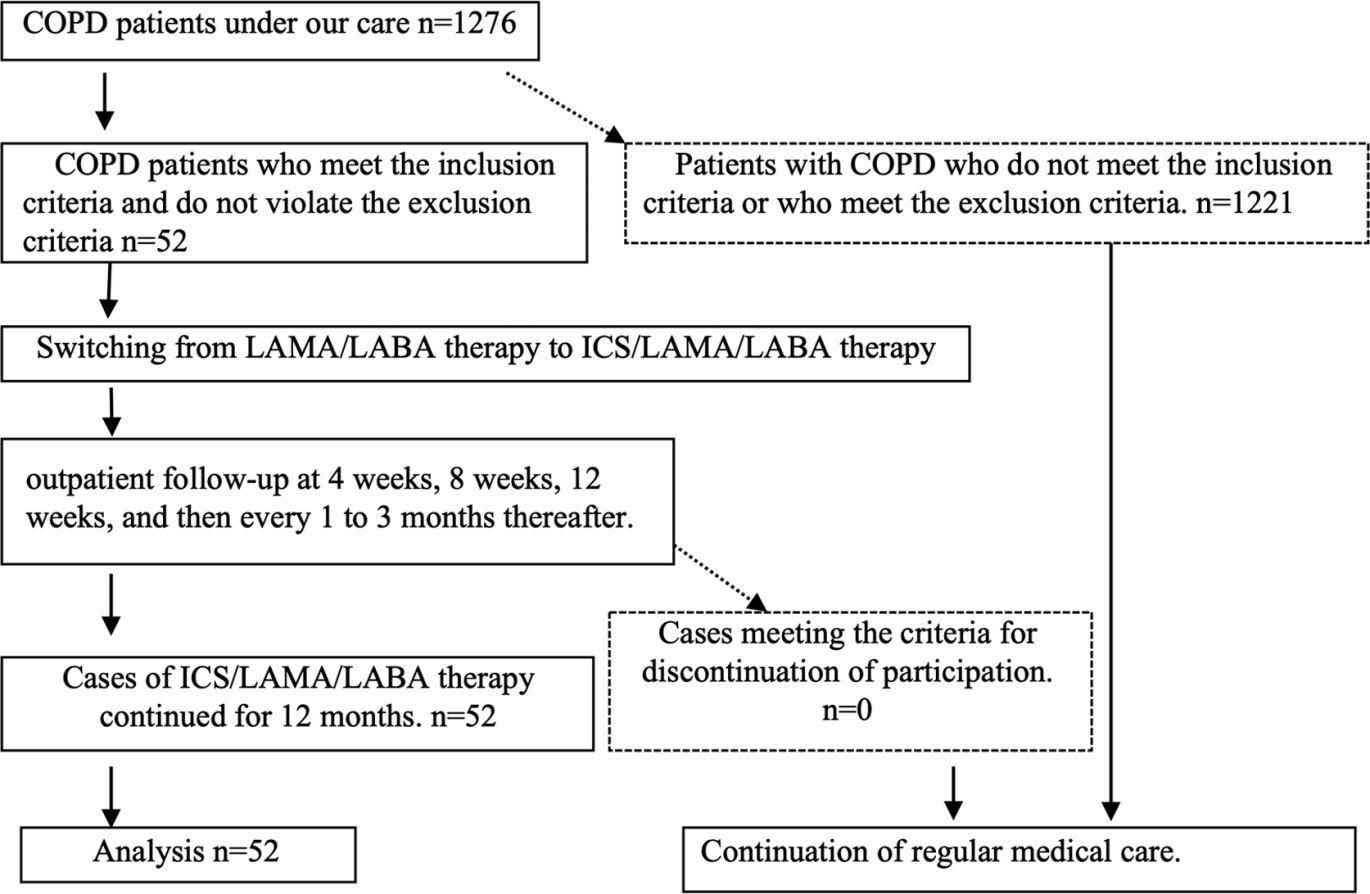
COPD management flow: Of a total of 1276 COPD patients, 52 met the inclusion criteria and did not meet any exclusion criteria. All 52 patients participated in the study, and their data obtained from them were analyzed.

In total, 52 patients with COPD who had been receiving LAMA/LABA therapy for ≥ 1 year and whose treatment was switched to ICS/LAMA/LABA therapy between April 2021 and April 2023 were included in the study (Figure 1 and Table 1).

**TABLE 1:** Changes in relevant parameters after switching from LAMA/LABA to ICS/LAMA/LABA therapy in patients with COPD.

	At entry	At 48 weeks	Mean difference	*p*‐value
Pulmonary function test				
FEV1 (L)	1.28 ± 0.60	1.33 ± 0.65	+0.045 ± 0.020	0.032*
FEV1/FVC (%)	46.69 ± 14.03	46.53 ± 16.68	−0.16 ± 0.69	0.81
%FEV1/predicted FEV1 (%)	56.89 ± 22.10	58.94 ± 23.46	+2.05 ± 0.87	0.023*
%VC	92.19 ± 19.62	94.00 ± 18.60	+1.78 ± 1.16	0.40
FVC (L)	2.70 ± 0.75	2.78 ± 0.75	+0.10 ± 0.041	0.017*
V50/V25	4.20 ± 1.19	4.09 ± 1.35	−0.10 ± 0.076	0.038*
Symptoms				
CAT scores	14.54 ± 9.53	13.52 ± 9.50	−1.02 ± 1.46	0.033*
mMRC scores	1.67 ± 1.23	1.52 ± 1.14	−0.15 ± 0.093	0.10
0/1/2/3/4(n)	11/15/8/16/2	12/15/12/12/1	—	0.78
IOS				
R5	3.54 ± 1.52	3.39 ± 1.46	−0.15 ± 0.076	0.052
Ex‐R5	3.95 ± 1.74	3.84 ± 1.72	−0.10 ± 0.078	0.19
In‐R5	3.13 ± 1.41	2.96 ± 1.31	−0.16 ± 0.096	0.096
R20	2.66 ± 0.94	2.58 ± 1.04	−0.079 ± 0.071	0.27
Ex‐R20	2.80 ± 1.01	2.76 ± 1.15	−0.048 ± 0.076	0.54
In‐R20	2.51 ± 0.92	2.39 ± 0.98	−0.12 ± 0.078	0.13
R5–R20	0.88 ± 0.71	0.83 ± 0.62	−0.053 ± 0.067	0.43
Ex R5–R20	1.14 ± 0.89	1.08 ± 0.81	−0.063 ± 0.062	0.31
In R5–R20	0.62 ± 0.64	0.58 ± 0.59	−0.044 ± 0.082	0.60
X5	−1.70 ± 1.59	−1.42 ± 1.23	+0.28 ± 0.14	0.059
Ex X5	−2.32 ± 2.34	−1.84 ± 1.79	+0.48 ± 0.21	0.028*
In X5	−1.05 ± 0.93	−1.00 ± 0.77	+0.046 ± 0.088	0.60
Fres	16.01 ± 6.67	13.91 ± 6.43	−2.11 ± 0.35	< 0.0001*
Ex Fres	18.28 ± 8.59	15.67 ± 7.89	−2.61 ± 0.43	< 0.0001*
In Fres	13.74 ± 5.57	12.14 ± 5.76	−1.60 ± 0.40	0.0002*
ALX	17.50 ± 15.97	8.28 ± 14.21	−9.20 ± 1.70	< 0.0001*
Ex ALX	11.17 ± 25.20	6.43 ± 24.21	−4.73 ± 1.00	< 0.0001*
In ALX	6.45 ± 8.28	2.15 ± 8.26	−4.30 ± 0.32	0.012*

*Note:* Data were presented as mean ± standard deviation. R5 reflects the total airway resistance, R20 reflects the resistance of relatively central airways, and R5–R20 reflects the resistance of peripheral airways; higher values reflect increased airway resistance. X5 reflects the frequency dependence of resistance. An increase in its absolute value in the negative direction represents an increase in elastic property. A decrease in Fres indicates a higher elasticity than the inertial property between two resistances.

Abbreviations: %VC, percent vital capacity/predicted vital capacity; ALX, low‐frequency reactance area; CAT, COPD Assessment Test; Ex ALX, low‐frequency reactance area in the expiratory phase; Ex Fres, frequency of resonance in the expiratory phase; Ex R5–R20, respiratory system resistance values at 5 Hz in the expiratory phase minus respiratory system resistance values at 20 Hz in the expiratory phase; Ex X5, reactance at 5 Hz in the expiratory phase; Ex‐R20, respiratory system resistance values at 20 Hz in the expiratory phase; Ex‐R5, respiratory system resistance values at 5 Hz in the expiratory phase; FEV1, forced expiratory volume in 1 s; Fres, frequency of resonance; In ALX, low‐frequency reactance area in the inspiratory phase; In Fres, frequency of resonance in the inspiratory phase; In R5–R20, respiratory system resistance values at 5 Hz in the inspiratory phase minus respiratory system resistance values at 20 Hz in the inspiratory phase; In X5, reactance at 5 Hz in the inspiratory phase; In‐R20, respiratory system resistance values at 20 Hz in the inspiratory phase; In‐R5, respiratory system resistance values at 5 Hz in the inspiratory phase; IOS, impulse oscillation system; mMRC, modified Medical Research Council; R20, respiratory system resistance values at 20 Hz; R5, respiratory system resistance values at 5 Hz; R5–R20, respiratory system resistance values at 5 Hz minus respiratory system resistance values at 20 Hz; V25, forced expiratory flow for 75% of FVC; V50, forced expiratory flow for 50% of FVC; VC, forced vital capacity; X5, reactance at 5 Hz.

All patients were either current (*n* = 10) or former smokers (*n* = 42), with a mean smoking history of 39.1 ± 23.4 pack‐years (range: 3–113). No never‐smokers were included.

Among these 52 patients (mean baseline %FEV1/predicted: 56.9% ± 22.1%), all had experienced one to two moderate exacerbations in the previous year despite LAMA/LABA therapy.

The baseline clinical characteristics (mean ± SD) were as follows: age, 72.56 ± 7.70 years; gender (male/female), 46/6; height, 162.46 ± 6.12 cm; weight, 57.13 ± 8.42 kg; FEV1, 1.28 ± 0.60 L; FEV1/FVC, 46.69% ± 14.03%; CAT score, 14.54 ± 9.53; and mMRC dyspnea scale score, 1.67 ± 1.23.

### 7.2 MostGraph (IOS)

**FIGURE 2:**
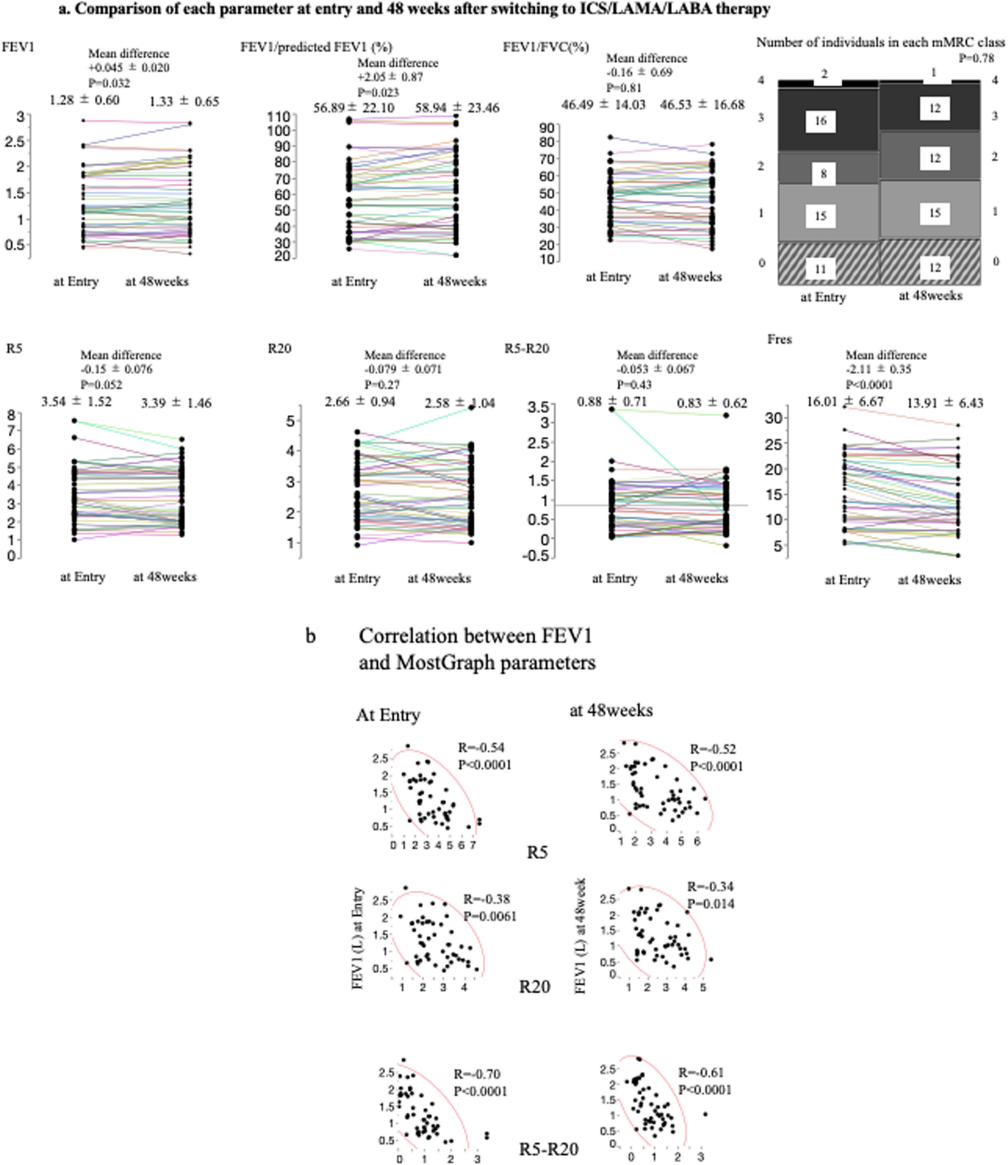
Changes and correlation between FEV1 and MostGraph parameters; data were presented as mean ± standard deviation. (a) Comparison of each parameter at entry and 48 weeks after switching to ICS/LAMA/LABA therapy. The airflow obstruction indicators FEV1 and FEV1/predicted FEV1 showed a significant difference between entry and 48 weeks; however, the difference was small and not clinically significant. There was no significant difference in FEV1/FVC, and the mMRC scores did not show a noticeable change. Among the MostGraph parameters, R5 (total airway resistance), R20 (central airway resistance), and R5–R20 (peripheral airway resistance) showed no significant differences between entry and 48 weeks. However, Fres, which reflects the ratio of inertial resistance to elastic resistance, was significantly lower at 48 weeks than at entry. (b) Correlation between FEV1 and MostGraph parameters. At entry and at 48 weeks, FEV1 showed a very strong correlation with all MostGraph parameters reflecting airway resistance (R5, R20, and R5–R20). FEV1, forced expiratory volume in 1 s; Fres, frequency of resonance; FVC, forced vital capacity; mMRC, modified Medical Research Council; R20, respiratory system resistance values at 20 Hz; R5, respiratory system resistance values at 5 Hz.

As shown in Table 1 and Figures 2a and S1, after switching from LAMA/LABA therapy to ICS/LAMA/LABA therapy, IOS parameters reflecting respiratory resistance, such as R5, R20, and R5–R20, as well as their values individually evaluated in the inspiratory and expiratory phases, did not change significantly. However, Fres significantly decreased (at entry: 16.01 ± 6.67 vs. at 48 weeks: 13.91 ± 6.43, mean difference −2.11 ± 0.35, *p* < 0.0001), which was also observed in the expiratory/inspiratory phases (Ex Fres: mean difference −2.61 ± 0.43, *p* < 0.0001/In Fres: mean difference −1.60 ± 0.40. *p* = 0.0002). These results are consistent with those of X5 in the expiratory phase (mean difference +0.48 ± 0.21, *p* = 0.028).

### 7.3 Pulmonary Function Test

There were no significant changes in the PFT parameters (Table 1 and Figure 2a).

Although there were significant differences in FEV1, %FEV1/predicted FEV1, and FVC before switching from LAMA/LABA to ICS/LAMA/LABA therapy and 48 weeks after switching, none of these changes were clinically significant.

In pulmonary function tests, all indicators of airway obstruction, such as FEV1, FEV1/predicted FEV1, and FEV1/FVC, demonstrated very good correlations with the respiratory resistance parameters obtained from MostGraph, including R5, R20, and R5–R20 (Figure 2b). Similarly, both the FEV1 measured at baseline and the FEV1 measured after 48 weeks showed good correlations with the respiratory resistance parameters obtained from MostGraph, whether measured during the expiratory or inspiratory phase (Figures S2 and S3).

### 7.4 Symptoms

The mMRC scores did not significantly change after switching from LAMA/LABA to ICS/LAMA/LABA. Although there was a significant change in the CAT scores after switching from LAMA/LABA to ICS/LAMA/LABA, the change was not clinically meaningful (Table 1 and Figure 2a).

### 7.5 Drug Safety and Adverse Event

Four patients required antibiotics for upper respiratory infections, but there were no adverse events necessitating the addition of steroids, emergency room visits, changes in treatment plans, or hospitalization.

None of the patients experienced moderate or severe COPD exacerbations requiring systemic corticosteroids or hospitalization during the 48‐week observation period.

Although spirometric and resistance parameters did not show significant changes following ICS addition, Fres significantly decreased, suggesting sensitivity to treatment‐related airway changes. Similarly, ALX, which also reflects the balance between elastic and inertial components, showed a consistent trend. In contrast, clinical indices such as CAT and mMRC scores remained largely unchanged. These findings imply that Fres and ALX may detect qualitative changes in airway mechanics not captured by spirometry or conventional resistance metrics.

## 8 Discussion

Pulmonary function test indicators (FEV1, FEV1/FVC, and FEV1/predicted) showed minimal changes 1 year after switching from LAMA/LABA to ICS/LAMA/LABA therapy, with no clinical significance. CAT and mMRC scores also exhibited only slight changes, consistent with previous reports. Similarly, IOS parameters measured using MostGraph (R5, R20, and R5–R20) showed no significant changes between baseline and 48 weeks postswitch.

The airway resistance indices obtained from MostGraph (R5, R20, and R5–R20) demonstrated a strong correlation with pulmonary function test indicators (FEV1, FEV1/FVC, and FEV1/predicted), emphasizing the close relationship between airway resistance and airflow obstruction in COPD. Among these, ExR5–ExR20, which reflects peripheral airway resistance during expiration, showed the strongest correlation with FEV1 at baseline (*R* = −0.66) and 48 weeks (*R* = −0.62). These findings align with the current understanding that COPD‐related airflow obstruction predominantly occurs in the peripheral airways during expiration. The results are consistent with our previous retrospective study [16].

In our earlier study, the switch from LAMA/LABA to ICS/LAMA/LABA therapy did not significantly affect airway resistance indices (R5, R20, and R5–R20), likely due to ICS's limited bronchodilatory effects. However, it significantly altered Fres, an index that reflects the ratio of inertial to elastic resistance, indicating an increase in elastic resistance. We hypothesized that these changes might reflect the anti‐inflammatory effects of ICS on airway remodeling, even though ICS has limited bronchodilatory effects. This interpretation is consistent with findings from the WISDOM trial, which reported no significant difference in time to exacerbation between patients who continued or discontinued ICS in triple therapy but observed a more rapid decline in lung function following ICS withdrawal [12].

This study was designed to confirm the reproducibility of these findings, and our results support these previous conclusions.

Importantly, although spirometric indices and clinical symptom scores (CAT, mMRC) did not show significant improvements, oscillometric indices such as Fres and ALX demonstrated notable changes. These indices reflect the balance between elastic and inertial resistance and may indicate qualitative changes in airway mechanics that are not captured by traditional parameters. This suggests that oscillometry may provide additional insights into airway behavior beyond conventional spirometry, particularly regarding the nature—not just the extent—of airflow limitation.

## 9 Limitations

This study has several limitations. First, the majority of participants were male, reflecting the higher smoking prevalence among men in Japan. This gender imbalance may limit the generalizability of the findings to female COPD patients. Second, the study predominantly included patients with mild COPD who had been receiving LAMA/LABA therapy for over a year without significant impairment to daily activities. Therefore, it remains uncertain whether these results apply to patients with more severe COPD or active airway inflammation. Third, the observed effects may be specific to the particular formulations of LAMA/LABA and ICS/LAMA/LABA used in this study, necessitating further investigations with the same formulations. Finally, the single‐arm design is a limitation of this study, as it lacks a control group for comparison.

Another limitation is that the hypothesis regarding ICS improving airway remodeling in COPD patients is based on assumptions derived from previous reports [12, 13, 16] and remains unsubstantiated. However, given the clinical benefits observed in prior studies of ICS intervention in COPD [12, 13, 16], it is plausible that ICS has some effect on COPD patients, potentially through suppressing airway remodeling. This possibility warrants further investigation.

Finally, this study was conducted during a period in which mask‐wearing was widely encouraged in Japan due to the COVID‐19 pandemic. Although spirometry and IOS assessments were conducted without masks, patients generally wore masks during daily activities. However, the extent and consistency of individual mask use could not be rigorously monitored, and its potential impact on infection exposure or airway inflammation during the study period remains uncertain.

## 10 Conclusions

Studies evaluating airway pathology in COPD using impulse oscillometry (IOS) with MostGraph are exceedingly rare. The airway resistance indices obtained from MostGraph demonstrated a strong correlation with traditional pulmonary function test indicators and exhibited excellent reproducibility. Additionally, as a noninvasive test that does not require forced deep breathing, MostGraph is well‐suited for evaluating airway pathology, particularly in elderly COPD patients.

Furthermore, Fres, a MostGraph‐derived index reflecting the balance between inertial and elastic resistance, showed consistent changes after switching to ICS/LAMA/LABA therapy. These changes suggest potential improvements in airway remodeling.

This study provides valuable insights and serves as a foundation for future research and the clinical application of MostGraph in COPD management.

## Author Contributions

All authors meet the following criteria: substantial contributions to the conception or design of the work; acquisition, analysis, or interpretation of data for the work; drafting the work or reviewing it critically for important intellectual content; final approval of the version to be published; and agreement to be accountable for all aspects of the work in ensuring that questions related to the accuracy or integrity of any part of the work are appropriately investigated and resolved. The final version of the manuscript has been reviewed and approved by all authors.

## Ethics Statement

This study protocol was reviewed and approved by the Medical Ethics Committee of Nippon Medical School, approval number [M‐2021‐033]. All participants provided a written informed consent.

## Conflicts of Interest

The authors declare no conflicts of interest.

## Supporting information


**Figure S1** Changes in exhalation and inhalation MostGraph parameters between at Entry and at 4 weeks after switching to ICS/LAMA/LABA. In both the exhalation phase (Ex) and inhalation phase (In), the values of R5, R20, and R5–R20 did not show significant differences between the values at entry and 48 weeks. However, significant differences were observed in Fres in both the exhalation phase (ExFres) and inhalation phase (InFres) when comparing the values at entry and 48 weeks.

**Figure S2** Correlation between FEV1 and MostGraph parameters in expiratory phase. The values of R5 (ExR5), R20 (ExR20), and R5–R20 (ExR5–ExR20) measured during the exhalation phase were significantly correlated with FEV1 measured both at entry and 48 weeks.

**Figure S3** Correlation between FEV1 and MostGraph parameters in inspiratory phase. The values of R5 (InR5), R20 (InR20), and R5–R20 (InR5‐InR20) measured during the inhalation phase were significantly correlated with FEV1 measured both at entry and at 48 weeks.

**Data S1** Supplementary Information.

**Data S2** Supplementary Information.

**Data S3** Supplementary Information.

**Data S4** Supplementary Information.

**Data S5** Supplementary Information.

**Data S6** Supplementary Information.

**Data S7** Supplementary Information.

**Data S8** Supplementary Information.

**Table S1** Schedule for the implementation of evaluation criteria (equivalent to routine general medical care performed at participating research institutions).

## Data Availability

The datasets generated and/or analyzed during the current study will be made available after the publication of the paper at [https://upload.umin.ac.jp/cgi‐open‐bin/ctr_e/ctr_view.cgi?recptno=R000042394] upon reasonable request from the corresponding author.
