# Supplementary figures and images for: Evaluation of the Effects of Switching COPD Patients From LAMA/LABA Therapy to ICS/LAMA/LABA Therapy Using the Impulse Oscillation System (IOS) Capable of Separating Inspiratory and Expiratory Measurements
Source: Clin Respir J. 2025 Jul 15;19(7):e70105. doi: 10.1111/crj.70105 (PMC12263508; doi:10.1111/crj.70105)

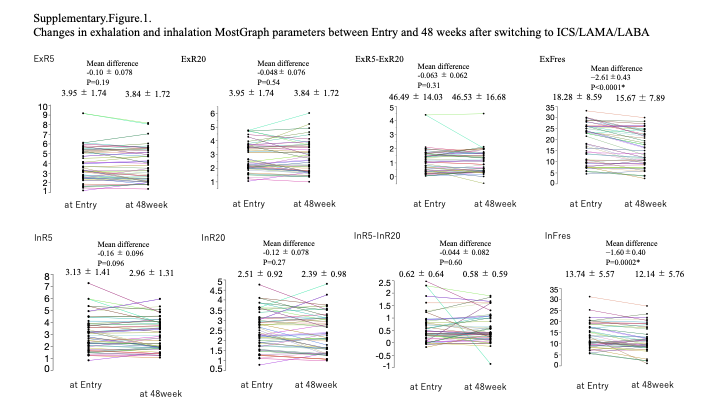

Supplement: Supplementary file 1 — Figure S1 Changes in exhalation and inhalation MostGraph parameters between at Entry and at 4 weeks after switching to ICS/LAMA/LABA. In both the exhalation phase (Ex) and inhalation phase (In), the values of R5, R20, and R5–R20 did not show significant differences between the values at entry and 48 weeks. However, significant differences were observed in Fres in both the exhalation phase (ExFres) and inhalation phase (InFres) when comparing the values at entry and 48 weeks. [file CRJ-19-e70105-s004.tiff]

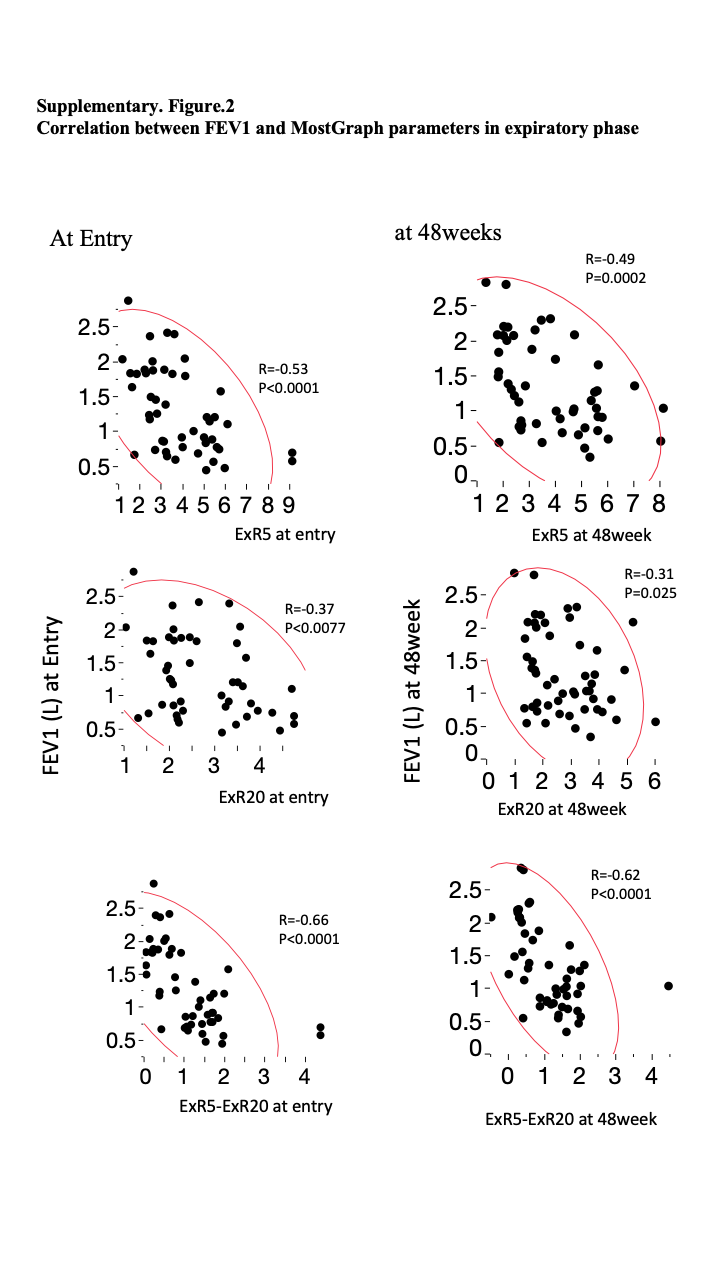

Supplement: Supplementary file 2 — Figure S2 Correlation between FEV1 and MostGraph parameters in expiratory phase. The values of R5 (ExR5), R20 (ExR20), and R5–R20 (ExR5–ExR20) measured during the exhalation phase were significantly correlated with FEV1 measured both at entry and 48 weeks. [file CRJ-19-e70105-s007.tiff]

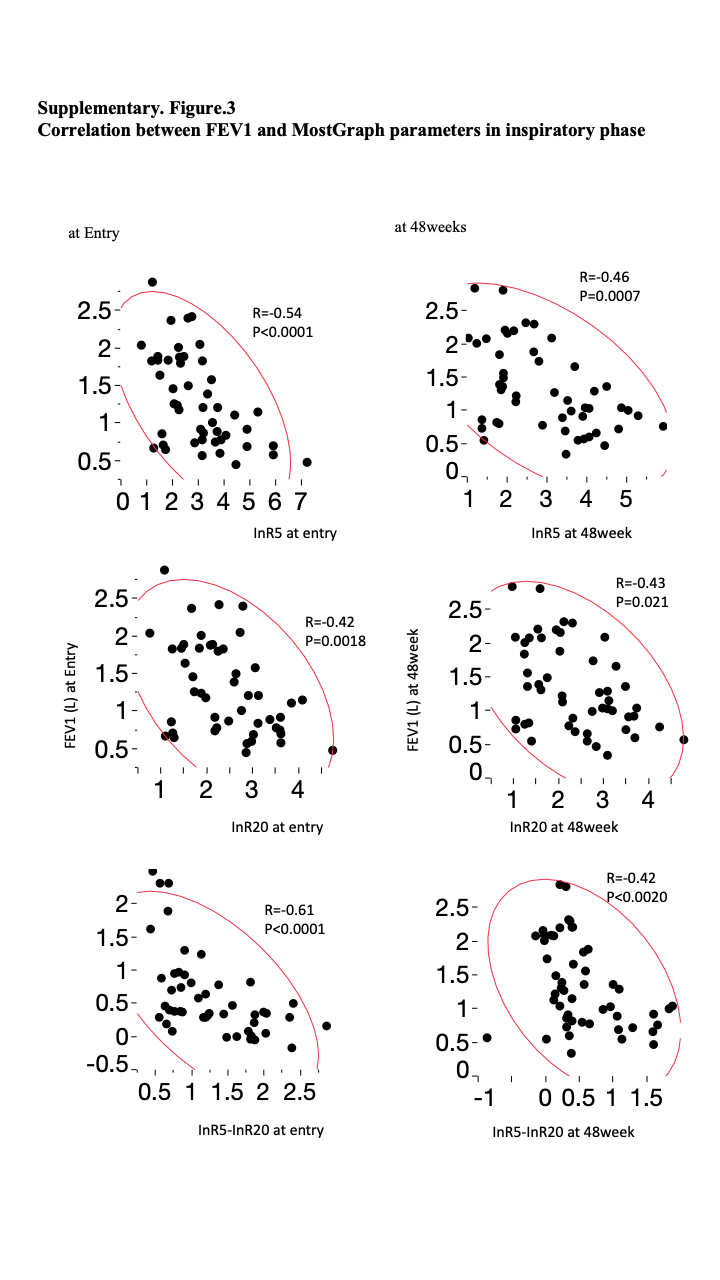

Supplement: Supplementary file 3 — Figure S3 Correlation between FEV1 and MostGraph parameters in inspiratory phase. The values of R5 (InR5), R20 (InR20), and R5–R20 (InR5‐InR20) measured during the inhalation phase were significantly correlated with FEV1 measured both at entry and at 48 weeks. [file CRJ-19-e70105-s011.tiff]
