# Supplementary material for: Evaluation of the Effects of Switching COPD Patients From LAMA/LABA Therapy to ICS/LAMA/LABA Therapy Using the Impulse Oscillation System (IOS) Capable of Separating Inspiratory and Expiratory Measurements
Source: Clin Respir J. 2025 Jul 15;19(7):e70105. doi: 10.1111/crj.70105 (PMC12263508; doi:10.1111/crj.70105)
Supplement: Supplementary file 4 — Data S1 Supplementary Information. [file CRJ-19-e70105-s005.docx]

**Supplementary file Handling of Adverse Events**

(i) Definition of Adverse Events

Adverse events are defined as undesirable changes observed or exacerbated during the observation period in the subject's signs, symptoms, conditions, events, or laboratory findings, regardless of their association with medication or treatment.

(ii) Definition of Serious Adverse Events

a. Those resulting in death

b. Those posing a threat to life

c. Those requiring hospitalization or prolongation of existing hospitalization for treatment

d. Those resulting in persistent or significant disability/incapacity

e. Those resulting in congenital anomalies

(iii) Recording of Adverse Events

In the event of an adverse event, the principal investigator or study collaborator will promptly provide appropriate treatment that can be performed within insurance-covered medical care, regardless of severity or frequency, record the event, and conduct follow-up investigations until the event disappears or returns to the state before the start of the study, as far as possible.

However, this does not apply to adverse events where causality is denied or adverse events associated with the worsening of the underlying disease or complications.

(iv) Reporting of Serious Adverse Events

In the event of a serious adverse event or the emergence of unpredicted new events, the principal investigator or study collaborator will provide appropriate treatment and promptly report to the hospital director and the Institutional Review Board for clinical trials.
