# Supplementary material for: Evaluation of the Effects of Switching COPD Patients From LAMA/LABA Therapy to ICS/LAMA/LABA Therapy Using the Impulse Oscillation System (IOS) Capable of Separating Inspiratory and Expiratory Measurements
Source: Clin Respir J. 2025 Jul 15;19(7):e70105. doi: 10.1111/crj.70105 (PMC12263508; doi:10.1111/crj.70105)
Supplement: Supplementary file 5 — Data S2 Supplementary Information. [file CRJ-19-e70105-s003.docx]

**Supplementary file "Assessment."**

Impulse Oscillation System (IOS) using MostGraph*

Pulmonary function tests: VC、%VC、FVC、%FVC、FEV1、FEV1%、TLC、RV、DLco、%DLco、DLco/VA、%DLco/VA

Blood tests (white blood cell count, hemoglobin, platelet count, AST (Aspartate Aminotransferase) ALT (, Alanine Aminotransferase), LDH (Lactate Dehydrogenase [), T-bil (Total Bilirubin [), creatinine (Cre), albumin (Alb), CK (Creatine Kinase) CRP (, C-Reactive Protein [), and D-dimer).

Imaging tests: Chest X-ray, Chest CT (assessment of COPD pathology and other findings)

Subjective symptoms, physical examination (height, weight), ADL (mMRC), etc.

Treatment information: Dates and doses of change in various medications, steroids, and other medications

Adverse events: Onset date, type, severity, outcome, outcome date

(Supplementary file Handling of Adverse Events. Supplementary file. Management of Adverse Events.)

Clinical events: Onset date, type, severity, outcome, outcome date

Prognostic information: Outcome, outcome date, cause of death

The above will be conducted from two weeks before the start date until the start

date and at 48 weeks, as well as needed for clinical necessity.

Primary endpoint: Longitudinal changes in MostGraph (indicative of total airway Resistance: R5, Respiratory system resistance values at 5 Hz; indicative of central airway resistance: R20, Respiratory system resistance values at 20 Hz; indicator of frequency-dependent resistance: X5, Reactance at 5 Hz; indicator of component of airway resistance: Fres, Frequency of resonance) 48 weeks after switching to ICS/LAMA/LABA therapy.

Secondary endpoints: Changes before and after each drug therapy using data, including cases discontinued during the trial with all indicators used for observing COPD pathology-related subjective symptoms and ADL (such as mMRC), pulmonary function tests, and all designated evaluation criteria.

Safety assessment: Blood tests as needed (white blood cell count, hemoglobin, platelet count, AST, ALT, LDH, T-bil, Cre, Alb, CK, CRP, and D-dimer); treatment information: dates and doses of various medication changes, steroids, and other medication status; adverse events: onset date, type, severity, outcome, outcome date; clinical events: onset date, type, severity, outcome, outcome date; prognostic information: outcome, outcome date, and cause of death.

Please refer to the supplementary file "Handling of Adverse Events" for details on the management of adverse events. (Supplementary file Handling of Adverse Events).

*MostGraph

Respiratory impedance was assessed using a commercially available oscillatory system (MostGraph-22 [Rev.1.2], Chest M.I., Co. Ltd., Tokyo, Japan), which has fulfilled standard recommendations, as described by Shirai et al. (15, 16). The cheeks of the participants were supported while sitting and wearing a nose clip. They were then instructed to breathe quietly at the functional residual capacity level (tidal breathing) for approximately 30 seconds. The measurements were repeated until five technically acceptable records were obtained. Respiratory impedance was automatically calculated via a fast Fourier transformation using a personal computer with airflow and pressure signals in the mouth of the participants. The respiratory system resistance values at 5 and 20 Hz (R5 and R20, respectively), difference between R5 and R20 (R5–R20), respiratory system reactance at 5 Hz (X5), Fres, and low-frequency reactance area (ALX) were evaluated. Each oscillatory index was expressed as the mean value during one respiratory cycle (whole breath), inspiratory and expiratory phases, and the difference between inspiratory and expiratory phases.

Eligible patients who had been receiving LAMA/LABA therapy for more than one year were switched from LAMA/LABA to ICS/LAMA/LABA within the period from the day all relevant parameters were evaluated two weeks later.

The evaluation 48 weeks after switching to ICS/LAMA/LABA therapy was conducted within the period from 48 to 49 weeks after the switch. Pulmonary function tests and impulse oscillometry (IOS) using MostGraph were performed when the study drug concentration was assumed to be at its trough level. Because MostGraph is a test conducted under normal breathing conditions, to avoid potential influences on the results of IOS, the IOS test using the MostGraph device was performed on the same day before the pulmonary function test.
