# Supplementary material for: Evaluation of the Effects of Switching COPD Patients From LAMA/LABA Therapy to ICS/LAMA/LABA Therapy Using the Impulse Oscillation System (IOS) Capable of Separating Inspiratory and Expiratory Measurements
Source: Clin Respir J. 2025 Jul 15;19(7):e70105. doi: 10.1111/crj.70105 (PMC12263508; doi:10.1111/crj.70105)
Supplement: Supplementary file 6 — Data S3 Supplementary Information. [file CRJ-19-e70105-s008.docx]

**Entry Criteria**

**Inclusion Criteria**

Patients aged 40 years or older (both male and female).

Patients diagnosed with COPD without other pulmonary diseases at our hospital, who had been receiving LAMA/LABA therapy for more than one year, had not experienced any significant COPD exacerbations in the past year, and had stable but persistent COPD symptoms that allowed for daily home activities.

Patients who were expected to not require changes to the treatment regimen of ICS/LAMA/LABA therapy for over one year.

Patients who were current or former smokers, with lung function showing respiratory system resistance values at FEV1% (Forced expiratory volume in 1 s (FEV1) / forced vital capacity (FVC)) <70%, and without diseases related to airflow obstruction other than COPD.

Patients were informed about the study using an informed consent document and provided consent to participate in this study.

**Exclusion Criteria**

1 Patients with a history of bronchial asthma.

2 Patients who show an increase in FEV1 of 200 ml or more before and after inhalation of a short-acting bronchodilator (400 μg salbutamol).

3 Patients with a peripheral blood eosinophil count ≥ 300 cells/μL.

4 Patients with asthma-like symptoms believed to be due to allergic factors.

5 Patients with serum IgE > 170 IU/mL.

6 Patients who have already been treated with any of the drugs LAMA, LABA, or ICS outside of this study.

7 Patients with diseases that cause respiratory symptoms other than COPD, such as heart disease leading to congestion.

8 Patients with closed-angle glaucoma.

9 Patients with urinary retention due to conditions such as prostate hypertrophy.

10 Patients with infections or deep-seated mycoses for which no effective antimicrobial agents are available.

11 Other patients whom the principal investigator or sub-investigator deems unsuitable for participation in this study.

Reason for Setting Exclusion Criteria

This study assessed the impact of switching from LAMA/LABA therapy to ICS/LAMA/LABA therapy on airway pathology in COPD using the IOS with MostGraph. Elements of bronchial asthma that responded positively to ICS were excluded based on the latest Asthma and COPD Overlap (ACO) guidelines, 2nd edition, proposed by the Japanese Respiratory Society (JRS).
