# Supplementary material for: Evaluation of the Effects of Switching COPD Patients From LAMA/LABA Therapy to ICS/LAMA/LABA Therapy Using the Impulse Oscillation System (IOS) Capable of Separating Inspiratory and Expiratory Measurements
Source: Clin Respir J. 2025 Jul 15;19(7):e70105. doi: 10.1111/crj.70105 (PMC12263508; doi:10.1111/crj.70105)
Supplement: Supplementary file 7 — Data S4 Supplementary Information. [file CRJ-19-e70105-s006.docx]

**Supplementary file IC and Details on Personal Information**

Procedure for Obtaining Informed Consent

Prior to registering study subjects, the principal investigator or study collaborator will provide the candidate subjects with an explanatory document approved by the Ethics Committee of Nihon Medical University Corporation, and conduct sufficient explanation both in writing and orally. Candidates will be given an opportunity to ask questions and sufficient time to decide whether to consent. Upon confirming that the candidate subjects have fully understood the content of the study, the principal investigator or study collaborator will obtain written consent from the candidate subjects based on their free will.

The tests and examinations conducted in this study correspond to the information obtained from regular clinical courses, but are performed for the purpose of analysis and consideration in the research objectives. As the accuracy of the analysis results is not guaranteed, disclosure of results to study subjects will not be conducted. However, if any findings are found that are considered to have a significant impact on the health or life of the study subjects, the disclosure of results will be discussed with the principal investigator, and individual responses will be provided accordingly.

7. Handling of Personal Information

Study subjects will be anonymized by assigning a study subject registration number, and only anonymized data that cannot identify individuals will be collected.

Personal information will be identified by the "medical record number" and "name" of the study subjects at the Department of Respiratory Medicine of Nippon Medical School Hospital and its affiliated facility, the Nippon Medical School Respiratory Care Clinic, where the study subjects are registered. Each subject will be assigned an anonymous "study subject registration number" using an anonymous correspondence table created for this study. The correspondence table that matches the study subject registration number with personal identification information (such as the study subject's medical record number, date of birth, etc.) will be kept by the safety management officer of the Department of Respiratory Medicine at Nippon Medical School Hospital on a password-protected personal computer (PC) not connected to the internet, ensuring safety measures against information leakage.

Researchers and related parties involved in this study have a duty of confidentiality concerning data handling and must make maximum efforts to protect personal information and privacy. Data obtained from the study subjects will not be used for any purpose other than the study.

If study subjects or their representatives withdraw their consent to continue the study, their personal information will be promptly discarded. Furthermore, in reporting and publishing the study results, information will not be disclosed in a manner that allows individual identification.
