# Supplementary material for: Evaluation of the Effects of Switching COPD Patients From LAMA/LABA Therapy to ICS/LAMA/LABA Therapy Using the Impulse Oscillation System (IOS) Capable of Separating Inspiratory and Expiratory Measurements
Source: Clin Respir J. 2025 Jul 15;19(7):e70105. doi: 10.1111/crj.70105 (PMC12263508; doi:10.1111/crj.70105)
Supplement: Supplementary file 8 — Data S5 Supplementary Information. [file CRJ-19-e70105-s009.docx]

**Supplementary file. Management of Adverse Events**

Management of Adverse Events

In the event of adverse events, the principal investigator or research collaborators promptly provide appropriate treatment that can be performed within the scope of insurance coverage, regardless of the severity or frequency, and record them. They will also conduct follow-up investigations until the events disappear or return to the pre-trial state, as much as possible.

However, this does not apply to adverse events where causality is ruled out, or adverse events associated with worsening of the underlying disease or comorbidities.

In cases of severe adverse events or unforeseen new events, the principal investigator or research collaborators will provide appropriate treatment and promptly report to the head of the research institution or the Central Ethics Committee of Nippon Medical School.
