# Supplementary material for: Evaluation of the Effects of Switching COPD Patients From LAMA/LABA Therapy to ICS/LAMA/LABA Therapy Using the Impulse Oscillation System (IOS) Capable of Separating Inspiratory and Expiratory Measurements
Source: Clin Respir J. 2025 Jul 15;19(7):e70105. doi: 10.1111/crj.70105 (PMC12263508; doi:10.1111/crj.70105)
Supplement: Supplementary file 9 — Data S6 Supplementary Information. [file CRJ-19-e70105-s010.docx]

**Supplementary file. Treatment Methods**

1. Investigational Treatment

Patients who had been on LAMA/LABA therapy for > 12 months were switched to ICS/LAMA/LABA therapy for 12 months.

2. Dosage and Administration of Investigational Treatment

The dosage and administration of each drug followed the instructions provided on the respective package inserts.

LAMA/LABA

Anoro Ellipta®

The usual dose for adults is one inhalation of Anoro Ellipta (containing 62.5 μg umeclidinium and 25 μg vilanterol) once daily.

Spiolto® Respimat®

For adults, the usual dose is two inhalations (containing 5 μg tiotropium and 5 μg olodaterol) once daily.

Ultibro® Breezhaler®

For adults, the usual dose is one capsule (containing 50 μg of glycopyrronium and 110 μg of indacaterol) once daily using a dedicated inhalation device.

The selection of LAMA/LABA agents (Anoro Ellipta®, Spiolto® Respimat®, or Ultibro® Breezhaler®) was determined by board-certified respiratory physicians based on each patient's inhalation capacity, handling ability, and clinical needs.

ICS/LAMA/LABA

Trelegy Ellipta® 100

The usual dose for adults is one inhalation of Trelegy Ellipta 100 (containing 100 μg of fluticasone furoate, 62.5 μg of umeclidinium, and 25 μg of vilanterol) once daily.

5) Treatment Period for Study Subjects

Study Period: April 15, 2021 – July 31, 2027

Enrollment Period: April 15, 2021 – July 31, 2026

Observation Period: 48 weeks from enrollment date of the last study However, enrollment is stopped once the target number of cases is reached.

Concomitant Therapy

Permissible Concomitant Medications:

Other therapeutic agents for underlying condition (COPD) besides ICS, LAMA, LABA

Other therapeutic agents besides ICS, LAMA, and LABA, such as antibiotics or oral steroids for exacerbations, or short-acting beta-agonists (SABA)

Prohibited Concomitant Medications: Investigational drugs
