# Supplementary material for: Evaluation of the Effects of Switching COPD Patients From LAMA/LABA Therapy to ICS/LAMA/LABA Therapy Using the Impulse Oscillation System (IOS) Capable of Separating Inspiratory and Expiratory Measurements
Source: Clin Respir J. 2025 Jul 15;19(7):e70105. doi: 10.1111/crj.70105 (PMC12263508; doi:10.1111/crj.70105)
Supplement: Supplementary file 11 — Data S8 Supplementary Information. [file CRJ-19-e70105-s001.docx]

**Supplementary file. Criteria for Termination of the Trial**

Criteria for Withdrawal of Study Participants:

Individual participants will be withdrawn from the study under the following circumstances:

(1) Withdrawal of consent from the study participants

(2) Identification of the need to discontinue the study drug owing to exacerbation of the underlying condition.

(3) Request the study participants to cease treatment modification.

(4) Pregnancy confirmed by the participants

(5) Other circumstances where the principal investigator or study collaborator deems the continuation of the study difficult

In case (1), the data obtained up to this point will not be utilized for the study.

For all other cases, the data obtained up to this point may be used for the study.

Criteria for Overall Termination of the Study:

The study may be terminated or suspended under the following circumstances:

Occurrence of serious adverse events

Termination of observation due to the death of a study participant.
