# Supplementary material for: Evaluation of the Effects of Switching COPD Patients From LAMA/LABA Therapy to ICS/LAMA/LABA Therapy Using the Impulse Oscillation System (IOS) Capable of Separating Inspiratory and Expiratory Measurements
Source: Clin Respir J. 2025 Jul 15;19(7):e70105. doi: 10.1111/crj.70105 (PMC12263508; doi:10.1111/crj.70105)
Supplement: Supplementary file 12 — Table S1 Schedule for the implementation of evaluation criteria (equivalent to routine general medical care performed at participating research institutions). [file CRJ-19-e70105-s002.docx]

Supplementary Table.1

Schedule for the implementation of evaluation criteria (equivalent to routine general medical care performed at participating research institutions).

| 項目 | | 開始前 | Day0 | 〜12week | | | 13week〜 | |
| --- | --- | --- | --- | --- | --- | --- | --- | --- |
| Time points | | Two weeks before to the Day0 | Day0 | Every 4weeks | At 12week | Every 4 to 12 weeks | | 48week |
| Obtaining consent | | ○ |  |  |  |  | |  |
| Confirmation of Patient Background | | ○ |  |  |  |  | |  |
| treatment with ICS/LAMA/LABA therapy | |  |  |  |  |  | |  |
| Confirmation of Self and Others' Symptoms | | ○ | ○ | ○ | ○ | ○ | | ○ |
| Observation of Adverse Events | |  |  |  |  |  | |  |
| Blood pressure measurement | | ○ | | as needed | ○ | as needed | | ○ |
| Pulse rate measurement | | ○ | | as needed | ○ | as needed | | ○ |
| Measurement of height and weight | | ○ | | as needed | ○ | as needed | | ○ |
| Clinical examination | Hematological examination | ○ | | as necessary | | | | ○ |
|  | Blood biochemical test | ○ | | as necessary | | | | ○ |
|  | Urinalysis | ○ | | as necessary | | | | ○ |
| Chest X-ray | | ○ | | as necessary | | | | ○ |
| ECG (electrocardiogram) | | ○ | | as necessary | | | | ○ |
| MostGraph | | ○ |  | as necessary | | | | ○ |
| ADL | | ○ |  |  | ○ |  | | ○ |
| Pulmonary function test | | ○ |  | as necessary | | | | ○ |

Assessment:

1. MostGraph (IOS)
2. Pulmonary function tests: VC, %VC, FVC, %FVC, FEV1, FEV1%, TLC, RV, DLco, %DLco, DLco/VA, %DLco/VA
3. Pulmonary function tests: Blood tests (WBC count, hemoglobin, platelet count, AST, ALT, LDH, T-bil, creatinine, albumin, CK, CRP, D-dimer)
4. Imaging tests: Chest X-ray, chest CT (COPD assessment and other findings)
5. Subjective symptoms, physical findings (height, weight), ADL (e.g., mMRC)
6. Treatment information: Dates and doses of medication changes, steroids, and other medications
7. Adverse events: Date of onset, type, severity, outcome, outcome date
8. Clinical events: Date of onset, type, severity, outcome, outcome date
9. Prognosis information: Outcome, outcome date, cause of death

These evaluations will be conducted according to the schedule outlined in the research methods.
